# Using cancer proteomics data to identify gene candidates for therapeutic targeting

**DOI:** 10.18632/oncotarget.28420

**Published:** 2023-05-04

**Authors:** Diana Monsivais, Sydney E. Parks, Darshan S. Chandrashekar, Sooryanarayana Varambally, Chad J. Creighton

**Affiliations:** ^1^Center for Drug Discovery, Baylor College of Medicine, Houston, TX 77030, USA; ^2^Department of Pathology and Immunology, Baylor College of Medicine, Houston, TX 77030, USA; ^3^Dan L. Duncan Comprehensive Cancer Center Division of Biostatistics, Baylor College of Medicine, Houston, TX 77030, USA; ^4^Cancer and Cell Biology Program, Baylor College of Medicine, Houston, TX 77030, USA; ^5^O’Neal Comprehensive Cancer Center, University of Alabama at Birmingham, Birmingham, AL 35233, USA; ^6^Molecular and Cellular Pathology, Department of Pathology, University of Alabama at Birmingham, Birmingham, AL 35233, USA; ^7^Genomic Diagnostics and Bioinformatics, Department of Pathology, University of Alabama at Birmingham, Birmingham, AL 35233, USA; ^8^The Informatics Institute, University of Alabama at Birmingham, Birmingham, AL 35233, USA; ^9^Human Genome Sequencing Center, Baylor College of Medicine, Houston, TX 77030, USA; ^10^Department of Medicine, Baylor College of Medicine, Houston, TX 77030, USA

**Keywords:** proteomics, proteogenomics, multi-omics, cancer, TTK protein kinase

## Abstract

Gene-level associations obtained from mass-spectrometry-based cancer proteomics datasets represent a resource for identifying gene candidates for functional studies. When recently surveying proteomic correlates of tumor grade across multiple cancer types, we identified specific protein kinases having a functional impact on uterine endometrial cancer cells. This previously published study provides just one template for utilizing public molecular datasets to discover potential novel therapeutic targets and approaches for cancer patients. Proteomic profiling data combined with corresponding multi-omics data on human tumors and cell lines can be analyzed in various ways to prioritize genes of interest for interrogating biology. Across hundreds of cancer cell lines, CRISPR loss of function and drug sensitivity scoring can be readily integrated with protein data to predict any gene’s functional impact before bench experiments are carried out. Public data portals make cancer proteomics data more accessible to the research community. Drug discovery platforms can screen hundreds of millions of small molecule inhibitors for those that target a gene or pathway of interest. Here, we discuss some of the available public genomic and proteomic resources while considering approaches to how these could be leveraged for molecular biology insights or drug discovery. We also demonstrate the inhibitory effect of BAY1217389, a TTK inhibitor recently tested in a Phase I clinical trial for the treatment of solid tumors, on uterine cancer cell line viability.

## INTRODUCTION

Gene expression profiling of human tumors can provide insights into the molecular subtypes of cancer and the pathways underlying more versus less aggressive disease [1–9]. Most of the gene expression data generated to date has been at the transcriptome level. However, recent technological advancements in mass spectrometry (MS)-based proteomics technologies have accelerated its application to study more and more human tumor specimens [10, 11]. MS-based proteomics can profile the expression of tens of thousands of protein features versus ~150–200 protein features typically involved in Reverse Phase Protein Arrays. Recent studies—e.g., from the Clinical Proteomic Tumor Analysis Consortium (CPTAC), the International Cancer Proteogenome Consortium (ICPC), the Applied Proteogenomics OrganizationaL Learning and Outcomes (APOLLO) Network, and others—have collectively made MS-based proteomic profiling data combined with corresponding multi-omics data (or “proteogenomic” data) on thousands of human tumors to date [10, 12, 13]. The initial proteogenomics studies that generated these data respectively studied the protein expression landscape of individual cancer types, including defining molecular subtypes at the protein level, determining the impact of somatic mutation on protein expression, and noting disparities of interest between mRNA and protein. The data from these studies have been put into the public domain for other research groups to explore them with new questions in mind, which may involve combining data from multiple individual studies.

The authors of this present Research Perspective have collectively participated in multiple pan-cancer proteomics studies, which have involved collecting and curating public MS-based proteomics data from multiple studies of diverse cancer types. With these data, we have defined pan-cancer proteomic subtypes of cancer [4, 6], explored the impact of somatic mutation on protein expression across diverse cancer types [4], defined gene correlates of pediatric brain tumor recurrence or progression at both mRNA and protein levels [5], and defined protein and mRNA correlates of tumor grade or stage for multiple cancer types [1]. Our study involving tumor grade correlates [1] would serve as a starting point for this Research Perspective, as this study involved both a bioinformatics component and a wet lab or bench experimental component. In this study, we mined the proteomic grade correlations, and we identified protein kinases—including MAP3K2, MASTL, and TTK—that by experiment had a functional impact *in vitro* in uterine endometrial cancer cells. For several reasons, studies that mine public cancer molecular datasets to identify novel gene targets for functional validation can be challenging, as, for example, there would be no obvious “best” approach to carry this out. Below, we consider some public molecular resources, including proteomics datasets, that may be leveraged to help identify gene candidates for therapeutic targeting in cancer.

### Proteomic grade correlations in tumors

Molecular signatures associated with clinical measures of advanced disease could provide molecular clues as to the drivers of more aggressive cancers [9]. Patient survival or time to adverse event would be one measure of aggressive disease. Time to cancer-specific death would perhaps be the preferable measure of patient survival, as it should be unambiguous. However, cancer-specific data requires a deliberate effort to follow up on the patient’s cause of death over time. Overall survival is much more commonly used in cancer studies and would serve as an adequate surrogate for cancer-specific death for most cancer types, though exceptions would include types of cancer such as prostate that tend to be more indolent [14]. Historically, tumors in big science multi-omics projects such as The Cancer Genome Atlas (TCGA) and CPTAC have involved less complete patient follow-up data [15], as the priority here was more for obtaining tumors with sufficient material for carrying out multiple assays on the same sample, even if the eventual disease course after initial surgery was unknown. This issue of lack of extensive patient follow-up involving the CPTAC datasets was something we faced in our study to identify proteomic correlates of aggressive cancer [1]. We, therefore, relied upon other surrogates for aggressive cancer, including tumor grade and stage. Cancer grade is a histologic parameter assigning the degree of differentiation of the cancer cells, where high-grade cancers look poorly differentiated and tend to grow and spread more quickly than low-grade cancers that look well-differentiated. Cancer stage is a clinical parameter indicating how extensively the tumor has spread outside of its site of origin. One might expect that high-grade versus high-stage tumors would tend to overlap, though the two represent different measures.

In our study, we sought to define differentially expressed proteins and mRNAs associated with higher grade or stage. We examined each of seven cancer types with MS-based proteomic data from CPTAC (breast, colon, lung adenocarcinoma, clear cell renal, ovarian, uterine, and pediatric glioma), representing 794 patients in total. For most cancer types, we found hundreds of protein features to be differentially expressed with higher grade or with higher stage. However, notably more statistically significant proteins were associated with higher grade than higher stage. For each cancer type, there was significant gene set overlap between the proteins and the corresponding mRNAs respectively associated with higher grade or higher stage. However, many genes significant at the protein level were not significant at the mRNA level and vice versa, indicative of widespread decoupling between the proteome and transcriptome. We could identify 1056 genes for which the total protein associated with grade in the same direction for two or more cancer types. At the same time, each cancer type showed a proteomic signature of tumor grade that was distinctive from the other cancer types. In surveying somatic copy number alterations associated with tumor grade, we found that proteins having lower expression with higher grade often involved genes more frequently lost at the copy number level with higher grade. Pathways of interest were enriched within the grade-associated proteins across multiple cancer types, including pathways of altered metabolism, Warburg-like effects, and translation factors.

The above results represent a useful exercise in bioinformatics and integrative analysis, providing some interesting insights along with a catalog of molecular correlates of aggressive disease. Still, we wanted to take these results a step further, to see if they might also drive experimental studies to identify gene targets in cancer. We hypothesized that protein correlates of higher tumor grade would include proteins having a functional impact beyond merely a correlative association. We examined the uterine data for potential targets for functional studies in uterine endometrial cell lines. We focused here on kinases, which tend to be more druggable [16, 17]. Taking a set of 347 protein kinases with available uterine tumor data, we found 37 associated with higher grade in uterine cancer, and 20 of these proteins were associated with grade in three or more cancer types studied (including uterine). From these 20 kinases, we selected four for functional studies: MAP3K2, MASTL, SCYL1, and TTK. We transfected Ishikawa and HEC-1-A cell lines with non-targeting siRNA or siRNA targeting each of these four kinase genes. Inhibition of TTK and MASTL resulted in decreased cell viability and migration *in vitro*. Inhibition of MAP3K2 decreased migration but not cell viability. In contrast, Ishikawa cells with SCYL1 knockdown demonstrated increased viability.

### Proteomics data portals

Additional proteins of interest remain to be uncovered and explored from the public proteomic datasets. Molecular biologists and physician-scientists need the tools to search these datasets independently without needing a bioinformatics expert. We have made a concerted effort to provide cancer proteomics datasets to the wider research community, in a way that facilitates a search for any gene of interest. Originally developed at the laboratory of Dr. Sooryanarayana Varambally, UALCAN (which stands for the University of ALabama at Birmingham CANcer data analysis portal) is a comprehensive, user-friendly, and interactive web resource for analyzing cancer -omics data [18, 19]. UALCAN (http://ualcan.path.uab.edu) allows users to analyze the relative expression of a query gene or genes across tumor and normal samples for a given cancer type, as well as in tumor sub-groups based on individual cancer stages, tumor grade, race, body weight, molecular and histologic subtypes, or other clinicopathologic features. The graphics provided by UALCAN, e.g., box plots comparing expression across tumor groups, can be output to a format amenable to incorporating into figures for publication or presentation (PNG, JPEG, SVG, or PDF).

**Figure 1:**
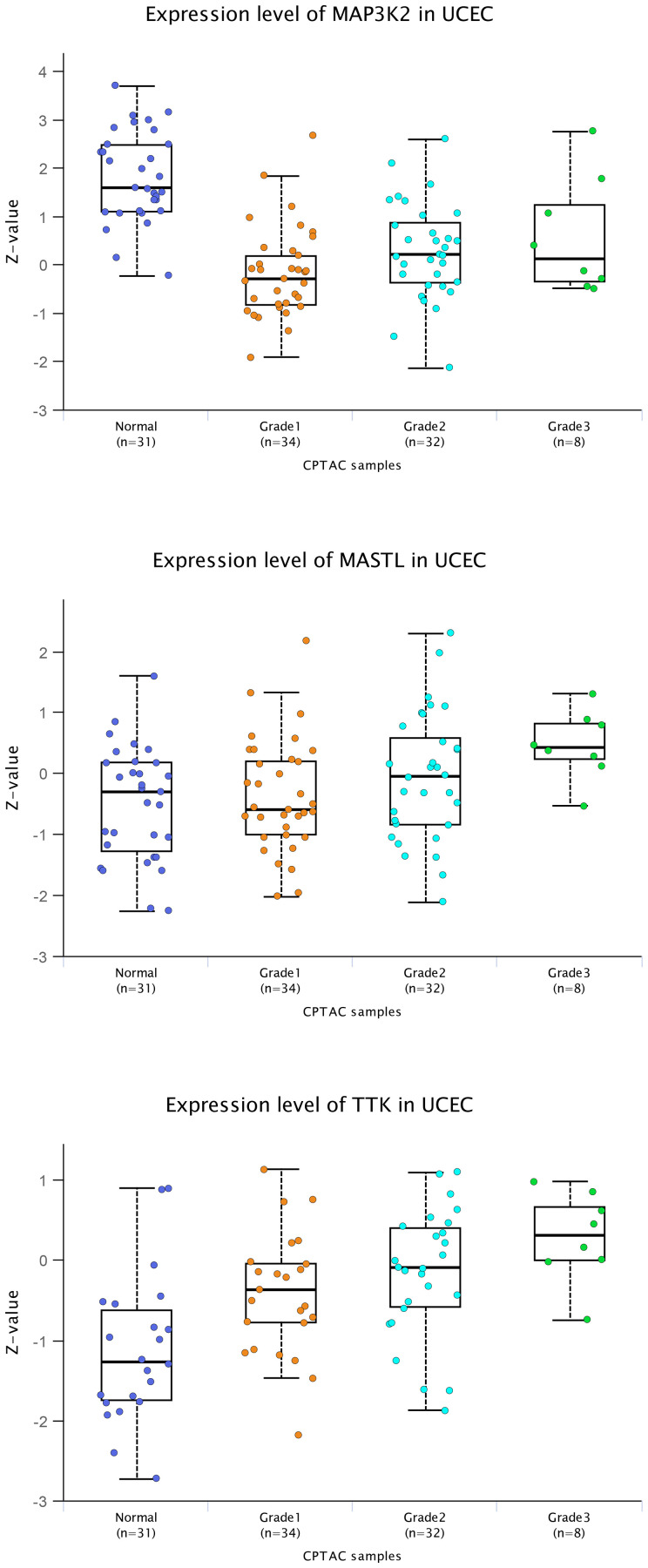
Association of selected proteins with higher grade in uterine tumors. MAP3K2, MASTL, and TTK were found in our recent study [1] to have functional impact *in vitro* in uterine endometrial cancer cells. The three genes were originally selected for study based on their protein expression association with higher tumor grade. Here, box plots of protein expression of these three genes illustrate the respective associations with tumor grade in human endometrial tumors, based on CPTAC data [24]. These box plots here were generated directly using the UALCAN data portal [18, 19], which provides publication-quality figures of gene-level views of expression datasets. Box plots represent 5% (lower whisker), 25% (lower box), 50% (median), 75% (upper box), and 95% (upper whisker). For each group, the actual number of samples with expression data for a particular protein may be fewer than the total number of samples in the dataset.

Initially, UALCAN housed transcriptomics data from TCGA, involving RNA-seq and clinical data from 31 cancer types [18]. Subsequently, we have incorporated MS-based proteomics data [1, 4, 6], primarily from CPTAC but including other sources [20–22]. We have also incorporated proteomic and transcriptomic data on pediatric brain tumors from the Children’s Brain Tumor Network (CBTN) into UALCAN [5, 23]. The user may input one or more genes of interest, then select clinicopathologic variables for group comparisons. Figure 1 shows box plots generated using UALCAN, representing the association of proteins MAP3K2, MASTL, and TTK with higher tumor grade in human endometrial tumors based on CPTAC data [24]. MASTL and TTK, associated with cell viability *in vitro*, have higher expression on average in cancer versus non-cancer tissues. In contrast, MAP3K2, which was not associated with cell viability, has lower expression in cancer versus non-cancer. Whether including a cancer versus normal comparison filter in addition to a tumor grade filter would help better refine a list of candidate gene targets would be an open question.

Data portals such as UALCAN would enable users to look up genes of interest to see if they show relevant differential or survival-associated expression patterns in human tumors. UALCAN results may complement results from experimental studies, providing evidence of the relevance of genes in the setting of human disease in addition to established relevance in model systems. Since 2017, UALCAN has been visited over one million times by cancer researchers from over 100 countries. Other commonly used data portals that house protein expression data along with other cancer -omics data include cBioPortal [25, 26]. However, there are notable differences between UALCAN and cBioPortal. cBioPortal mainly focuses on gene mutations and copy number alterations (CNA) data in cancer, with visualization capabilities mostly revolving around highlighting mutation and CNA patterns along with expression outliers for specific genes. Unlike UALCAN, with cBioPortal no global comparisons can be accomplished for tumor subgroups. While user-friendly data portals allow some level of access to molecular data from a wide audience of researchers who may not write code but can use “point-and-click” interfaces, there are limitations on the types of questions such tools can answer. There would remain a clear need for bioinformatics experts who can carry out high-level analyses and data integration to answer more sophisticated questions [27].

### Cancer cell line data

Notwithstanding their limitations, cell line model systems remain extremely useful for basic cancer research and drug discovery. While issues such as serial passaging and artificial growth conditions likely modify cancer cells grown *in vitro* over time to some degree, authenticated cancer cell lines retain most of the genetic properties of the cancer of origin [28, 29]. Cell lines can provide the preliminary data for many projects to justify further exploration using more sophisticated experimental models, including organoids and patient-derived xenografts (PDXs). For hundreds of cancer cell lines, there are extensive molecular and perturbation data available in the public domain from major endeavors, including the Cancer Cell Line Encyclopedia (CCLE) [30, 31] and the Genomics of Drug Sensitivity in Cancer (GDSC) [32, 33]. For PDX tumors, concerted efforts involving the NIH-NCI PDX Development and Trial Centers Research Network (PDXNet) and the NIH-NCI Patient-Derived Models Repository (PDMR) repositories have recently generated multi-omic data on over 1500 PDX tumors to date representing over 500 patients [2, 34]. However, proteomics data on these tumors is currently limited, and perturbation data (e.g., gene knockdowns or drug treatments) remain to be systematically generated [35].

**Figure 2:**
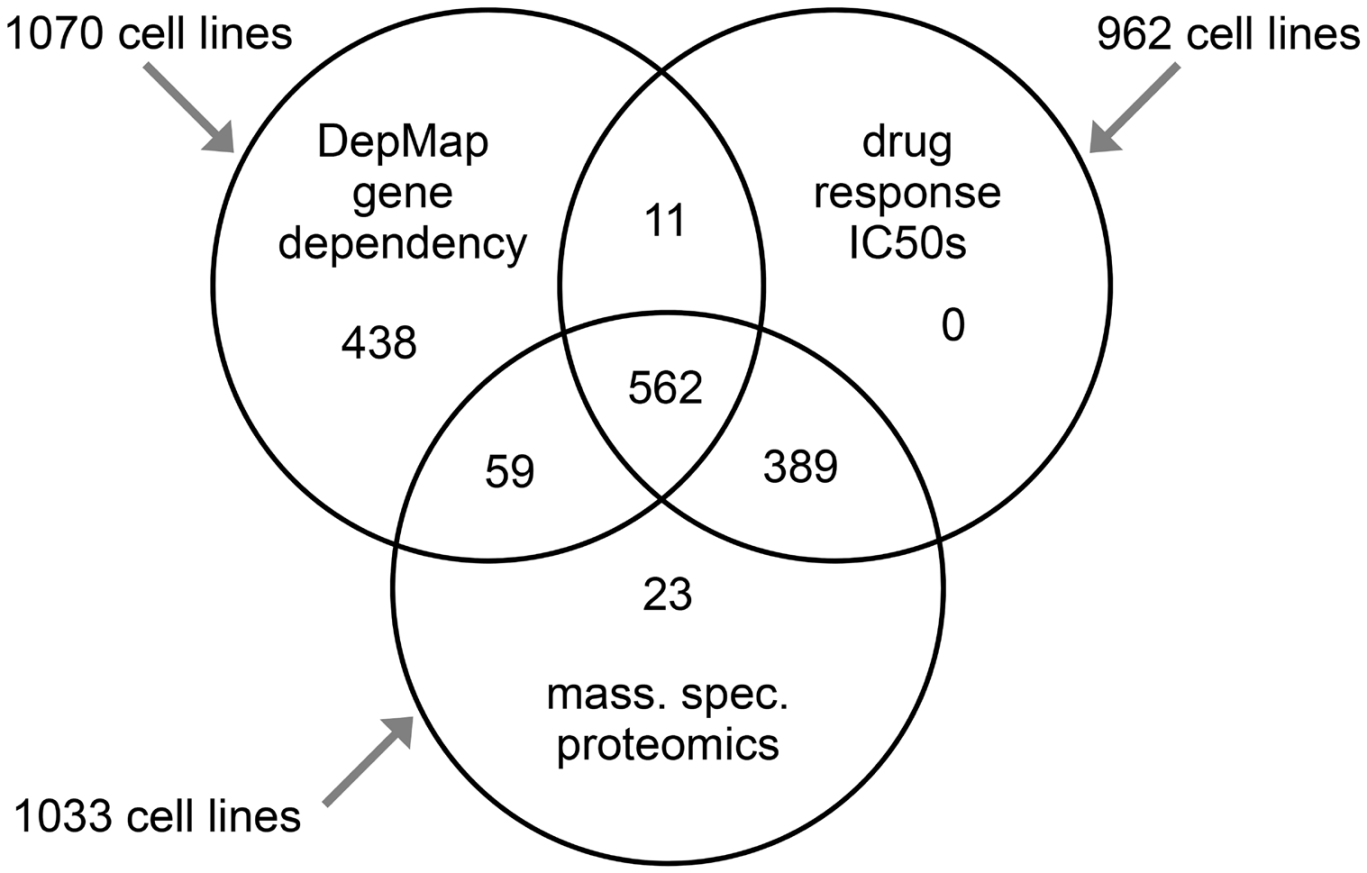
Compiled multilevel data on cancer cell lines. Venn diagram shows the number of cell lines with data involving protein expression (by mass spectrometry), CRISPR assays, and drug sensitivity. Gene effect scores, based on Cancer Dependency Map (DepMap) CRISPR assays, were taken from the dataset as analyzed using the Chronos algorithm from Dempster et al. [38]. We compiled the mass spectrometry-based proteomics data on 949 cell lines in total from Gonçalves et al. [37] and on 375 cell lines in total from Nusinow et al. [36]. For any proteomic values not represented in the Gonçalves dataset (e.g., missing values or cell lines not represented), we used the values from Nusinow. We then z-normalized protein expression values to standard deviations across cell lines in the combined dataset. We downloaded Genomics of Drug Sensitivity in Cancer (GDSC) drug compound half maximal inhibitory concentration (IC50) data in February 2020 (GDSC1-dataset) and in October 2022 (GDSC2-dataset) [32, 33, 37]. We merged the two GDSC IC50 datasets into one. If a drug treatment and cell line were represented in both datasets, we averaged the two values; otherwise, we used whichever IC50 dataset had available data. GDSC IC50 data represented 623 drug treatments involving 544 compounds.

Multilevel data on cancer cell lines can be brought together from different sources and studies for integrative analyses. CCLE datasets comprise extensive multi-omics data on over 1000 cancer cell lines, with data platforms including whole-exome sequencing, whole-genome sequencing, DNA methylation data by reduced representation bisulfite sequencing, metabolomics, and proteomics by Reverse Phase Protein Array [30]. On the other hand, GDSC involves much more extensive drug response data than CCLE, where GDSC includes half maximal inhibitory concentration (IC50) data across hundreds of cancer cell lines for 523 drug compounds. In contrast, CCLE includes IC50 data for some two dozen compounds. MS-based proteomics data have been generated for both CCLE and GDSC cell lines, involving 378 cell lines of the former [36] and 949 cell lines of the latter [37]. The Cancer Dependency Map project, or DepMap, has carried out large-scale CRISPR loss of function screens on over 1,000 cancer cell lines. DepMap data allow for inferring gene knockout fitness effects for any given gene in each cell line, based on an explicit model of cell proliferation dynamics after CRISPR gene knockout [38, 39]. Figure 2 shows the numbers of cell lines respectively represented in the MS-based proteomics, CRISPR assays, and drug sensitivity datasets, as well as cell lines shared between datasets. As hundreds of cell lines would be represented between any two datasets, this should provide robust statistical power for integrative analyses, e.g., between protein expression and gene knockout fitness or drug responses across cell lines.

### CRISPR screens in cell lines

**Figure 3:**
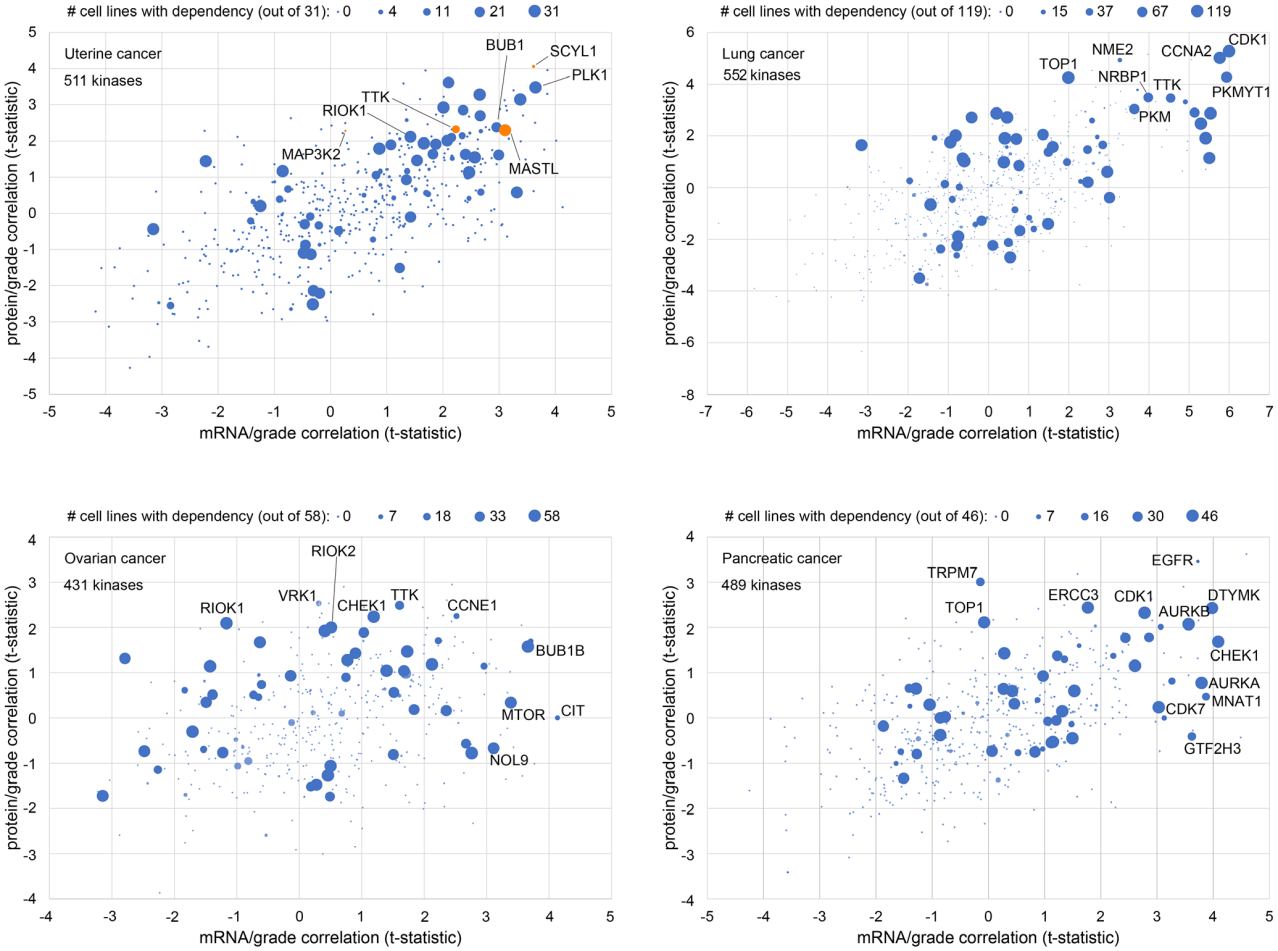
Combined analysis of kinase expression in tumors with cell line dependency to identify new gene targets. For each of four different cancer types (uterine, lung, ovarian, pancreatic), protein kinase abundance correlates with tumor grade are plotted against corresponding mRNA expression correlates. T-statistics either greater than 2 (higher with high grade) or less than −2 (lower with low grad) would be statistically significant (*p* < 0.05, Pearson’s correlation). Points are sized according to the number of cell lines of the given cancer type with a dependency by CRISPR assay, according to DepMap [38, 39]. DepMap scores of <−0.75 were called as denoting sensitivity of the given cell line for the given gene. The uterine cancer panel highlights the four genes explored in downstream functional experiments in our recent study [1].

In our recent study [1], in which we selected four kinases for functional studies based on tumor proteomics data, we did not incorporate perturbation data from cell lines into our selection of gene targets. Here, in Figure 3, we consider how gene dependency data by CRISPR in cell lines might be integrated with gene expression in human tumors. For each of four different cancer types (uterine, lung, ovarian, pancreatic), we plotted protein kinase abundance correlates with tumor grade against corresponding mRNA expression correlates. In addition, the data points in each of the four scatterplots are sized according to the number of cell lines of the given cancer type with a dependency by CRISPR assay, according to DepMap. Gene dependency indicates that the given cell line is vulnerable to knockdown of that gene. The methodology by Dempster et al. [38] effectively corrects for several biases and artifacts that can confound the raw DepMap results.

From Figure 3, we see that, between different cancer types, different sets of genes may be significantly correlated with tumor grade at the protein or mRNA levels. Genes significantly correlated with tumor grade at the protein level may not be significant at the mRNA level and vice versa, which warrants our integrative approach to incorporating both levels of data. The Figure 3 uterine cancer scatterplot highlights the four kinase genes we explored in downstream functional experiments [1]. Three of these four kinases had a corresponding mRNA association with grade, while MAP3K2 did not. In addition, we see that for each cancer type, most of the kinase genes examined had few or no cell lines dependent on that gene, though kinase genes significantly correlated with higher grade tended to be dependent for a high percentage of cell lines. Notably, MAP3K2 and SCYL1, which kinases did not yield optimal results *in vitro* for representing gene targets [1], have almost no uterine cell lines with corresponding DepMap dependency. For other genes, however, most cell lines in the DepMap dataset show a dependency. In this case, the question would arise as to whether the gene would represent a dependency only for cancer but not normal cells. In contrast, genes dependent for only a subset of cell lines (e.g., *TTK*) might represent better therapeutic targets, as these could involve uniquely targetable dependencies for a subset of cancers, e.g., genes involved in “oncogene addiction” [40–42].

### Drug targeting of genes

**Figure 4:**
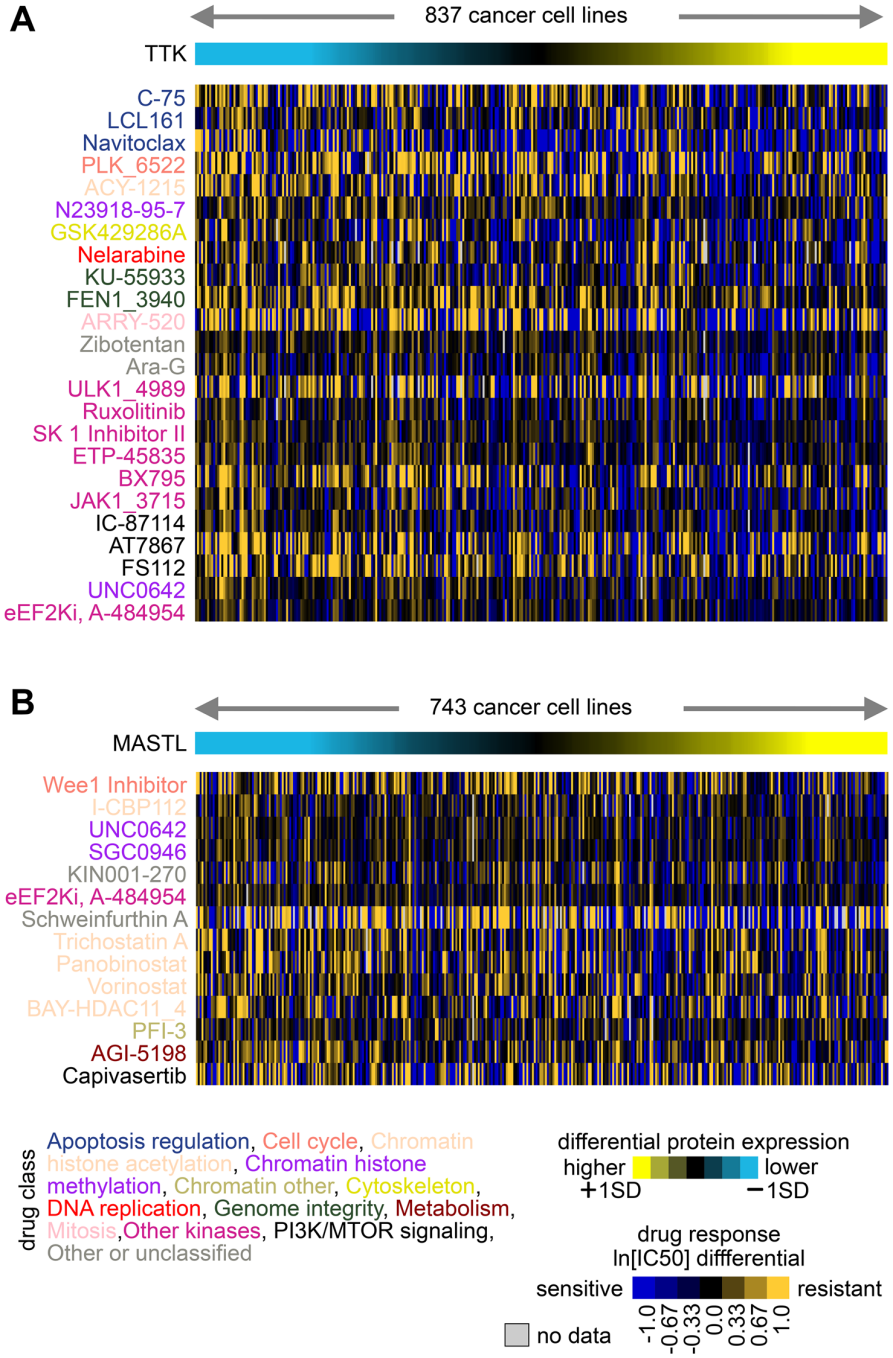
Associations of TTK and MASTL protein expression with drug responses across cancer cell lines. (**A**) From the GDSC [32, 33] cell lines with combined protein and drug response data, top compounds with decreases in IC50 associated with TTK protein expression (*p* < 0.001, one-sided Pearson’s correlations using natural log transformed IC50 values). Drug compound names are colored by drug class. (**B**) Similar to part A, but for top compounds with decreases in IC50 associated with MASTL protein expression (*p* < 0.01, one-sided Pearson’s correlations).

Cancer cell lines that have been extensively characterized and assayed for their sensitivity to a large collection of pre-clinical and clinical therapeutic agents might enable therapeutic biomarker discovery [29]. Gene expression data can be integrated with drug IC50 data to identify markers of drug response [2, 37, 43]. Using a dataset of combined protein expression with drug IC50s involving 544 compounds and 621 treatments (Figure 2), we looked for associations of protein expression with drug responses across cancer cell lines, involving the three kinase genes—MAP3K2, MASTL, and TTK—that we studied *in vitro* in uterine cancer cell lines [1]. A negative correlation between IC50 values and protein expression indicates that cell lines with higher expression tend to be most sensitive to the drug. With 544 compounds considered, we might expect about five and less than one to have nominally significant *p*-values of <0.01 and <0.001, respectively, due to multiple testing [44]. Interestingly, MASTL, which had fewer cell lines with protein data (*n* = 255), had no significant IC50 associations exceeding chance expected. However, both TTK and MASTL each had numerous drug response associations exceeding chance expected, as highlighted in Figure 4. For example, TTK expression correlates with increased sensitivity to several kinase inhibitors, while MASTL expression correlates with increased sensitivity to several drugs targeting chromatin histone acetylation. These results would provide additional information as to which existing drugs might target cancers that over-express a particular marker, perhaps even using a combinatorial treatment strategy.

**Figure 5:**
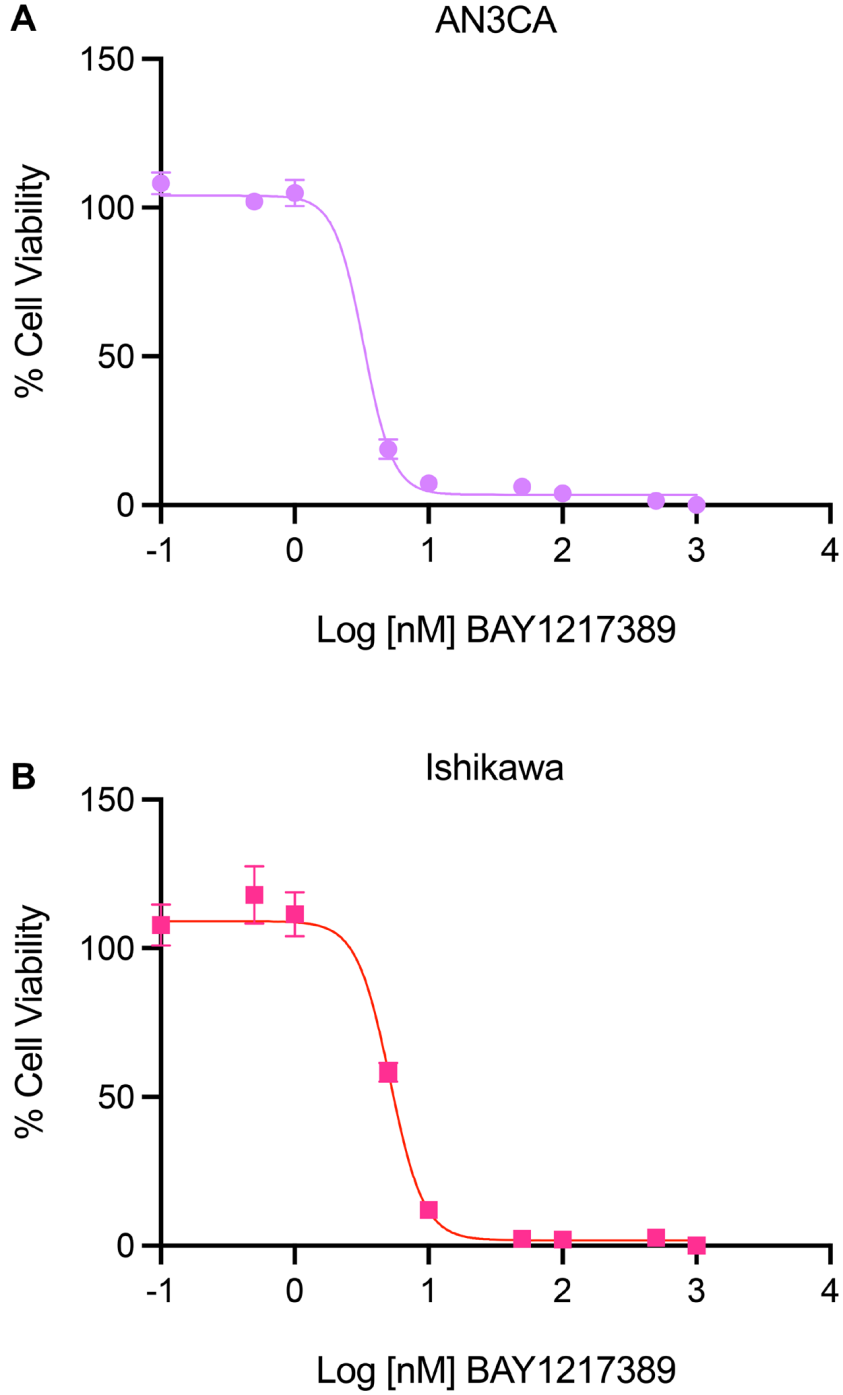
Efficacy of TTK inhibitor, BAY1217389, in AN3CA and Ishikawa uterine cancer cell lines. (**A**, **B**) The effect of TTK inhibitor, BAY1217389, on the viability of endometrial cancer cell lines, AN3CA (A) and Ishikawa (B), was evaluated by quantifying intracellular ATP concentrations using a luminescence-based assay (CellTiter GLO, Promega). 72 hours after incubation with various concentrations of the TTK inhibitor, BAY1217389, the IC50 values of this compound were found to be 3.276 nM in AN3CA and 5.166 nM in Ishikawa cells. This graph represents data from three repeated trials, analyzed by a non-linear regression model with a four-parameter variable slope (Y = Bottom + (Top-Bottom)/(1+10^((LogIC50-X) × HillSlope)) (GraphPad Prism, version 9).

Here, to validate the biological relevance of the overlap between gene dependency, drug response, and mass spectrometry proteomics data, we tested the effect of BAY1217389, a TTK inhibitor recently tested in a Phase I clinical trial for the treatment of solid tumors [45, 46], on uterine cancer cell line viability *in vitro*. In these assays, AN3CA and Ishikawa uterine cancer cells were plated in a 96-well plate (1,000 cells/well). The following day, the cells were treated in triplicate with various concentrations of BAY1217389. After 72 hours, cell viability was assessed by quantifying cellular ATP via luminescence using CellTiter Glo. The IC50 values of BAY1217389 in AN3CA and Ishikawa cells were 3.276 nM and 5.166 nM, respectively (Figure 5). The potency of this TTK inhibitor suggests that targeting TTK is biologically relevant in uterine cancer cells and provides rationale for further testing in patient-derived organoid systems or animal models of endometrial cancer.

In addition to using existing drugs to target gene function in cancer, there is the potential to discover new drugs to target genes of interest. In recent studies [47–50], the Drug Discovery Center at Baylor College of Medicine has identified novel active and specific inhibitors using DNA-Encoded Chemistry Technology (DEC-Tec), including, more recently, potent and selective molecules that inhibit the kinase activity of BMPR2 [50]. DEC-Tec allows the exploration of chemical space at a greater level than traditional high-throughput screening methods [51]. Drug discovery programs with DEC-Tec operate on the premise of encoding small organic molecule compounds (~10^8^ per library) with individual and unique DNA tags, to identify novel small-molecule inhibitors [52]. In DEC-Tec selections, the affinity-tagged recombinant protein targets are incubated with DNA-encoded molecules in the presence or absence of a competitive ligand. Target binders are captured using antigen-coated magnetic beads with affinity to the recombinant protein tag. Bound molecules are eluted by denaturing the protein, and the DNA barcodes of recovered library material are then PCR amplified and submitted for Next Generation Sequencing. Library members with a high affinity for the target will be retained at a higher rate during the screen and will consequently be present at enriched concentrations in the sequenced DNA pool. Candidate hit molecules are identified by decoding the DNA tags. Candidates with favorable physiochemical properties can then be synthesized without the DNA tag and tested *in vitro*. Using DEC-Tec or other drug discovery platform, one might identify novel small-molecule inhibitors of kinase genes in uterine cancer, including TTK and MASTL, representing future work from our previous study [1].

### Multiple approaches for selecting targets

Our above study examining proteomic correlates of tumor grade [1] represents just one approach in utilizing public molecular datasets to select gene targets. There would be no single integrative analysis approach to identifying gene targets of interest. Numerous datasets and gene selection criteria may be utilized, including associations with parameters of more aggressive disease, cancer versus “normal” comparisons (though there may be issues in finding a suitable normal [53, 54]), DNA mutation or CNA patterns, and molecular patterns in experimental model systems such as cell lines or mice. Molecular datasets considered may involve a single cancer type or multiple cancer types. Selecting genes for further study often involves close collaboration between a bioinformatician and an experimentalist. Ideally, the experimentalist would be actively involved in the selection process. In practice, it often helps for the bioinformatician to provide the experimentalist a top set of genes meeting one or more selection criteria, from which the experimentalist can pick a few genes for preliminary studies (e.g., *in vitro* experiments). Domain knowledge of molecular biology should factor into the decision process over merely relying on statistical cutoffs alone. Below, we provide additional examples from previous studies, with one component analyzing molecular profiling data on human tumors and another component of bench experimental follow-up based on the analysis results. However, we cannot provide a comprehensive overview here of all cancer-related studies using this broad approach.

In ovarian cancer, we have been involved in studies of microRNAs (miRNAs) [53, 55, 56]. miRNAs are ~22 nt noncoding RNAs which target complementary gene transcripts for translational repression or mRNA cleavage [57]. In comparing both mRNA and miRNAs for serous ovarian tumors and cell lines with normal cells, we identified miR-31 as an under-expressed miRNA deleted at the copy number level in an appreciable number of serous ovarian tumors represented in TCGA. In subsequent experiments, miR-31 over-expression *in vitro* inhibited proliferation and induced apoptosis in a number of cell lines [53]. mRNA and miRNA expression profiling of clear cell ovarian cell lines identified miRNAs of interest, including miR-100, which we found in follow-up experiments to inhibit mTOR signaling and enhance sensitivity to mTOR inhibitor everolimus [56]. In another study that surveyed correlations between miRNAs and their predicted mRNA targets across over 400 serous ovarian cancers in TCGA database, the miR-29 family and predicted targets were among the top results. Subsequent experiments showed that over-expression of miR-29a *in vitro* repressed several anti-correlated genes, including *DNMT3A* and *DNMT3B*, and decreased cancer cell viability [55].

The senior author of this Research Perspective has participated with others in lung cancer studies, where we successfully utilized molecular profiling data of experimental models to identify gene targets [58–62]. In a seminal study of epithelial-mesenchymal transition (EMT) [61], analysis of molecular data from tumor cell lines derived from mice that develop metastatic lung adenocarcinoma identified the miR-200 family as differentially expressed. Subsequently, forced expression of miR-200 abrogated the capacity of these tumor cells to undergo EMT, invade, and metastasize, and conferred transcriptional features of metastasis-incompetent tumor cells. In another series of studies [58–60], bioinformatics analysis of molecular profiling data, involving cross-species comparison of the genes overexpressed in autochthonous genetically engineered metastatic murine lung tumors and syngeneic lung cancer models, intersected with human copy number amplifications by TCGA, identified a set of 217 putative driver genes in lung cancer [58]. Subsequent experiments have demonstrated functional roles for several of these genes, including *GATAD2B* [58], *TMEM106B* [59], *IMPAD1* [60], and *KDELR2* [60]. In another study, from analysis of TCGA CNA datasets, we identified a chromosome 1q region frequently amplified in diverse cancer types and encoding multiple regulators of secretory vesicle biogenesis and trafficking, including the Golgi-dedicated enzyme phosphatidylinositol (PI)-4-kinase IIIβ (PI4KIIIβ). Extensive follow-up experiments demonstrated PI4KIIIβ as a therapeutic target in chromosome 1q-amplified lung adenocarcinoma [62].

As evidenced by the thousands of literature citations to date of the seminal paper originally introducing the UALCAN data portal to the research community [18], UALCAN has facilitated the selection of genes for experimental validation for perhaps hundreds or even thousands of independent studies. The UALCAN creators themselves have utilized the data portal in several experimental studies of individual genes—including *PAICS* [63–66], *MTHFD1L* [67], *PAK4* [68], *P4HA1* [69], and *FZD8* [70]. In these studies, the relevance of the gene in human disease is first demonstrated by UALCAN analysis of a particular cancer type (e.g., showing higher expression in cancer versus normal, or specific cancer sub-class pattern such as ERG gene fusion specific over-expression of *FZD8* in prostate cancer [70]), followed by validation and a demonstration of the functional role of that gene in cancer cell models.

## CONCLUSIONS

As bench experiments can represent a great deal of effort, the ability of molecular profiling data to drive the selection of genes and the design of bench experiments could save much time and result in less trial-and-error. MS-based proteomic data can capture gene expression information at the protein level that would not be captured at the mRNA level [4, 10, 71]. UALCAN and other data portals can facilitate access to proteomic and other molecular datasets, providing gene-level results to guide future studies. UALCAN currently houses human tumor data, though the extensive molecular datasets on cancer cell lines might eventually be incorporated into UALCAN as well. At the same time, point-and-click interfaces are limited in terms of what questions they can answer [27]. Skilled bioinformaticians could integrate molecular data from multiple sources and at multiple -omics levels, including proteomics. Creative analytical approaches to the public cancer molecular datasets could yield new gene sets representing new therapeutic targets and new insights into cancer.

## Abbreviations

CPTAC: Clinical Proteomic Tumor Analysis Consortium
TCGA: The Cancer Genome Atlas
CBTN: Children’s Brain Tumor Network
CNA: copy number alterations
DEC-Tec: DNA-Encoded Chemistry Technology
MS: mass spectrometry
PDX: patient-derived xenograft
CCLE: Cancer Cell Line Encylopedia
GDSC: Genomics of Drug Sensitivity in Cancer
IC50: half maximal inhibitory concentration
